# Isosorbide dinitrate as an effective adjunct therapy for acute urinary retention: A randomized controlled trial

**DOI:** 10.1002/bco2.70252

**Published:** 2026-07-27

**Authors:** Salman Soltani, Hamid Reza Ghorbani, Mahmoud Tavakkoli, Fatemeh Sadabadi, Seyyed Parham Ahmadi, Farid Zeinali

**Affiliations:** ^1^ Kidney Transplantation Complications Research Center Mashhad University of Medical Sciences Mashhad Iran; ^2^ Department of Urology Mashhad University of Medical Sciences Mashhad Iran

**Keywords:** 5α‐reductase inhibitor, acute urinary retention, benign prostatic hyperplasia, isosorbide dinitrate, randomized clinical trial, α1‐blocker

## Abstract

**Objective:**

To evaluate the synergistic effect and safety of sublingual isosorbide dinitrate (ISDN) to facilitate successful voiding trial after catheterization for acute urinary retention (AUR) secondary to benign prostatic hyperplasia (BPH).

**Material and methods:**

In this randomized clinical trial, male patients aged ≥ 50 years presenting with first‐episode AUR secondary to BPH at our urology emergency department were enrolled during 2019–2020. Participants were randomly assigned to receive either standard therapy (α1‐blocker and 5α‐reductase inhibitor) alone (*n* = 40) or in combination with daily sublingual ISDN 10 mg for 21 days (*n* = 40). Primary outcomes included successful voiding post‐catheter removal as trial without catheter (TWOC) and post‐void residual (PVR) volume. Secondary outcomes included post‐treatment prostate‐specific antigen (PSA) levels and adverse events. Data were analysed using independent *t*‐tests and chi‐square tests.

**Results:**

A total of 80 men with similar baseline characteristics were enrolled (mean ages: 70.3 ± 7.97 in the ISDN group vs. 68.2 ± 8.73 years in the controls). The rate of successful TWOC was significantly higher in the ISDN plus standard therapy group compared with standard therapy alone (50.0% vs. 22.5%, *p* = 0.011). PVR volume decreased significantly after treatment in both groups (within‐group *p* < 0.001), with a greater overall reduction observed in the ISDN group (*p* = 0.002). PSA levels remained stable, with no significant within‐ or between‐group differences. On multivariable logistic regression analysis, adjunctive ISDN therapy independently predicted successful TWOC (adjusted OR 3.27, 95% CI 1.23–8.70; *p* = 0.017). No serious adverse events were observed.

**Conclusions:**

Adjunctive sublingual ISDN significantly improves urinary outcomes following AUR and reduces residual urine volume without increasing adverse events. This combination therapy offers a safe and effective strategy to improve the success of TWOC in patients with BPH‐related AUR.

## 1 INTRODUCTION

Acute urinary retention (AUR) represents the most prevalent urological emergency, defined as the inability to voluntarily void despite sustained effort, according to the International Continence Society.^1^ The lifetime risk of AUR rises substantially with age, affecting up to 10% of men in their 70s and nearly one‐third of men in their 80s.^2^ The male‐to‐female incidence ratio has been estimated at around 13:1, reflecting a gender disparity.^3^ The condition not only causes significant discomfort and distress but also predisposes patients to urinary tract infection, renal dysfunction and recurrent retention if inadequately managed.^4^ The underlying mechanisms contributing to AUR are multifactorial, involving bladder outlet obstruction, neurological dysfunction and detrusor muscle impairment.^5^

Benign prostatic hyperplasia (BPH), the leading cause of AUR in men, is a non‐malignant enlargement of the prostate that can obstruct the bladder outlet and impair urinary flow.^6^ BPH represents a substantial global health burden, with 2021 estimates reporting more than 13 million incident cases and an age‐standardized prevalence exceeding 2700 per 100 000 population.^7^ Its prevalence rises sharply with age—from roughly 15% at 40 years to nearly 37% at 80 years—and over one‐quarter of men worldwide are affected, with up to 80% experiencing BPH‐related symptoms by age 70.^8^, ^9^

Management of AUR typically requires prompt decompression through catheterization, followed by medical intervention to facilitate spontaneous voiding and prevent recurrence.^3^ Standard medical therapy to facilitate a successful trial without catheter (TWOC) primarily targets the dynamic component of obstruction. Alpha‐1 adrenergic antagonists (e.g., tamsulosin) relax smooth muscles in the prostate and bladder neck, whereas 5‐alpha reductase inhibitors (e.g., finasteride) reduce prostate volume and the risk of recurrent AUR.^10^, ^11^, ^12^ Despite these therapies, a subset of patients fails TWOC and may continue to require prolonged catheterization or surgical intervention, highlighting the need for alternative or adjunctive therapeutic strategies to improve bladder emptying more effectively.^13^

Nitric oxide (NO) has emerged as a key modulator of lower urinary tract physiology, functioning as a principal inhibitory neurotransmitter in non‐adrenergic, non‐cholinergic (NANC) pathways.^14^, ^15^, ^16^, ^17^ It promotes urethral relaxation through activation of soluble guanylate cyclase (sGC) and subsequent accumulation of cyclic guanosine monophosphate (cGMP) in smooth muscle cells. This pathway has been shown to facilitate smooth muscle relaxation in the genitourinary tract, potentially reducing intraluminal urethral pressure and facilitating micturition.^15^, ^16^, ^17^, ^18^, ^19^, ^20^ Pharmacologic NO donors, such as isosorbide dinitrate (ISDN), present a mechanistically rational novel therapeutic strategy.^21^

Preliminary clinical evidence has begun to explore this hypothesis. Sublingual ISDN significantly increased the rate of immediate spontaneous voiding among men presenting with AUR secondary to BPH, with 30% of patients voiding successfully compared with 3% with placebo.^22^ In contrast, a subsequent double‐blind trial assessing oral extended‐release ISDN in combination with tamsulosin demonstrated a numerically higher, but not statistically significant, improvement in TWOC success rates (81.6% vs. 67.5%).^23^ These disparate results may reflect differences in drug formulation, dosing regimen or timing of administration. Moreover, the safety profile of ISDN in this clinical context remains insufficiently characterized.

Taken together, the available evidence suggests that ISDN may represent a promising adjunctive therapy in the management of AUR, although its efficacy and safety profile have not been conclusively established. To address these gaps, the present randomized clinical trial was designed to evaluate the therapeutic potential of sublingual ISDN as an adjunct to standard medical therapy in men presenting with AUR due to presumed BPH. Specifically, this study aimed to determine whether ISDN could increase TWOC success rates and improve post‐void residual (PVR) volumes while maintaining an acceptable safety profile.

## 2 METHODS

### 2.1 Study design and setting

This prospective, randomized, controlled clinical trial was conducted at the Urology Emergency Department of Imam Reza Hospital, Mashhad, Iran, between 2019 and 2020. The trial aimed to evaluate the efficacy and safety of adjunctive sublingual ISDN in patients with AUR secondary to BPH.

### 2.2 Patient selection

Male patients aged ≥ 50 years presenting with a first episode of AUR presumed to result from BPH were eligible. Inclusion criteria comprised: (1) first episode of AUR; (2) age > 50 years; (3) absence of concurrent pharmacologic therapy or surgical indication; (4) confirmed BPH‐related obstruction. Patients were excluded if they had: (1) refusal to provide informed consent; (2) significant cardiac, hepatic, renal or metabolic comorbidities; (3) febrile illness (T > 38°C); (4) abnormal prostate‐specific antigen (PSA > 4 ng/mL) or suspicious digital rectal examination suggesting malignancy; (5) alternative causes of retention such as neurogenic bladder.

### 2.3 Randomization and blinding

Participants were randomly assigned in a 1:1 ratio to either standard therapy alone (tamsulosin 0.4 mg orally once daily plus finasteride 5 mg orally once daily) or standard therapy combined with adjunctive ISDN (10 mg sublingually once daily) for 21 days. Randomization was performed using a computer‐generated sequence in Microsoft Excel, with allocation concealed using sequentially numbered codes in sealed envelopes (A or B). Investigators responsible for outcome assessment were blinded to group allocation.

### 2.4 Interventions

All patients underwent baseline assessments, including ultrasonography, PSA measurement and digital rectal examination, to rule out malignancy or other pathological conditions. Foley catheterization was performed for all patients at baseline. The standard therapy group received tamsulosin and finasteride as above, whereas the intervention group additionally received 10 mg sublingual ISDN daily. No dose adjustments were made during the study period. After 21 days, Foley catheters were removed in the outpatient setting. Participants were instructed to report any adverse events throughout the study period.

### 2.5 Definitions and outcomes

The primary outcome was successful TWOC, defined as spontaneous voiding within 24‐h post‐catheter removal with PVR volume < 150 mL (measured by bladder ultrasonography). Secondary outcomes included: (1) changes in PVR volume from baseline to 21 days, (2) changes in PSA levels from baseline to 21 days and (3) incidence of drug‐related adverse events. All data were recorded using a structured checklist. Follow‐up assessments were conducted at 24‐h post‐catheter removal and at 21 days after initiation of therapy.

### 2.6 Sample size

Sample size was determined based on an anticipated increase in TWOC success rates from 30% in the standard therapy group to 60% with adjunctive ISDN, using *α* of 0.05 and 80% power. This calculation required 35 participants per group. To account for potential 10% attrition, 39 patients per group were recruited.

### 2.7 Statistical analysis

Data were analysed using SPSS Version 24 (IBM Corp., Armonk, NY, USA). Continuous variables were expressed as mean ± standard deviation (SD), and categorical variables as counts and percentages. Between‐group comparisons were performed using independent‐samples *t*‐tests for continuous variables and chi‐square tests for categorical variables. Within‐group changes were analysed using paired *t*‐tests as appropriate. A repeated measures analysis of variance (ANOVA) was used to evaluate changes in PVR over time and to compare these changes between groups. Time (pre‐intervention and post‐intervention) was treated as the within‐subject factor, and group (case vs. control) was included as the between‐subject factor. The time × group interaction term was used to assess whether the magnitude of change in PVR over time differed between the two groups, which represents the primary effect of interest in pre–post intervention studies. To identify factors associated with successful TWOC, a two‐step regression approach was employed. First, univariate binary logistic regression analyses were conducted using candidate variables of age, disease duration, baseline PVR, baseline PSA and treatment group. Variables demonstrating a *p*‐value < 0.20 in univariate analysis were subsequently entered into a multivariable binary logistic regression model. Results of logistic regression analyses are reported as odds ratios (ORs) with 95% confidence intervals (CIs). A *p*‐value < 0.05 was considered statistically significant.

### 2.8 Ethical considerations

The study protocol received approval from the Institutional Review Board of Mashhad University of Medical Sciences (Ethics Committee IR.MUMS.MEDICAL.REC.1398.643) and registered with the Iranian Registry of Clinical Trials (IRCT20170417033489N5, https://irct.behdasht.gov.ir/trial/47587). Written informed consent was obtained from all participants prior to enrollment. All procedures were conducted in accordance with the Declaration of Helsinki.

## 3 RESULTS

### 3.1 Participant characteristics

Eighty patients were enrolled (40 per group) (Figure S1). Baseline demographic and clinical variables, including age, disease duration, PVR volume and PSA, were comparable between groups, with no significant differences observed (all *p* > 0.05; Table 1).

**TABLE 1:** Baseline demographic and clinical characteristics of study participants.

Variable	Standard therapy (controls, *n* = 40)	ISDN + standard therapy (cases, *n* = 40)	*p*‐value
Age (years)	68.2 ± 8.7	70.3 ± 8.0	0.265
Disease duration (days)	127 ± 73.4	144.4 ± 64.5	0.861
PVR (mL)	1112.0 ± 156.8	1075.6 ± 182.8	0.342
PSA (ng/mL)	2.1 ± 1.0	2.1 ± 0.9	0.790

*Note*: Data are presented as mean ± standard deviation. Comparisons between groups were performed using the independent samples *t*‐test.

Abbreviations: ISDN, isosorbide dinitrate; PSA, prostate‐specific antigen; PVR, post‐void residual.

### 3.2 Clinical outcomes

Adjunctive ISDN therapy significantly improved TWOC success rate compared with standard therapy alone (50.0% vs. 22.5%; *p* = 0.011). Patients receiving ISDN accounted for 68.9% (*n* = 20/29) of successful TWOC cases, whereas TWOC failure occurred more frequently in the control group (60.8%, *n* = 31/51). This association was statistically significant (*χ* ^2^ = 6.55, Table 2).

**TABLE 2:** Clinical outcomes, post‐void residual volume (PVR) and prostate‐specific antigen (PSA) before and after treatment in patients receiving standard therapy alone or ISDN plus standard therapy.

Variable		Standard therapy (controls, *n* = 40)	ISDN + standard therapy (cases, *n* = 40)	*p*‐value*
TWOC success rate		9 (22.5%)	20 (50%)	**0.011**
PVR (mL)	Pre‐treatment	1112.0 ± 156.8	1075.6 ± 182.8	**0.002**
	Post‐treatment	171.6 ± 72.3	82.7 ± 9.8	
	Within‐group *p*‐value**	**< 0.001**	**< 0.001**	
PSA (ng/mL)	Pre‐treatment	2.10 ± 1.0	2.10 ± 0.9	0.766
	Post‐treatment	2.11 ± 0.93	2.04 ± 0.89	
	Within‐group *p*‐value**	**0.002**	**0.002**	

*Note*: Values are presented as number (percentage) or mean ± SD. Significant *p*‐values (<0.05) are visualized in bold.

Abbreviations: ISDN, isosorbide dinitrate; PSA, prostate‐specific antigen; PVR, post‐void residual; TWOC, trial without catheter.

* *p*‐values based on the chi‐square test or between‐group comparisons (case vs. controls) of repeated measures analysis of variance (ANOVA).

** *p*‐values based on within‐group comparisons (pre‐ vs. post‐treatment) of repeated measures analysis of variance (ANOVA).

**FIGURE 1:**
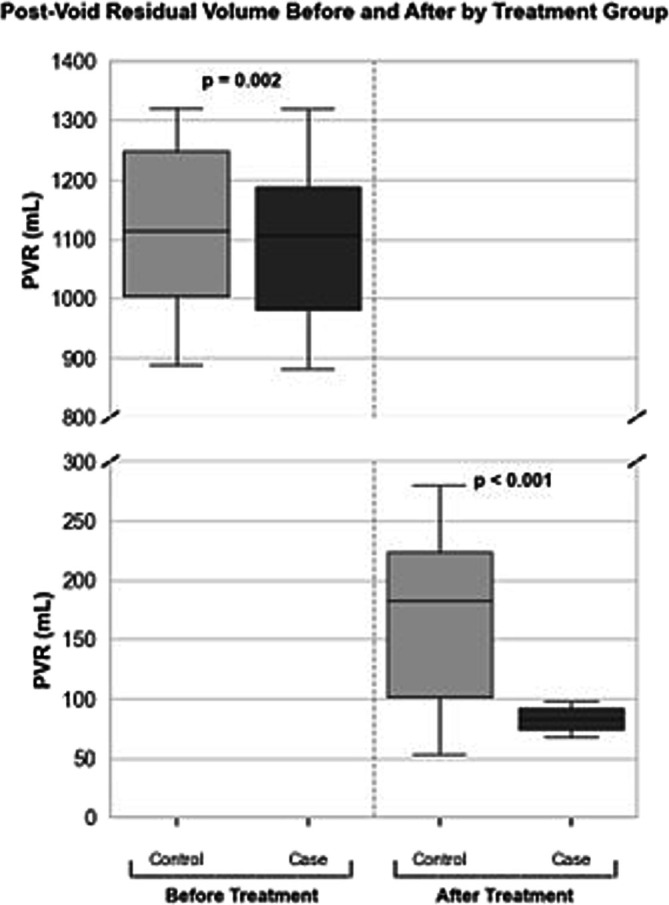
Changes in post‐void residual volume before and after treatment in control and ISDN groups.

PVR decreased significantly in both groups following treatment (within‐group *p* < 0.001). However, the magnitude of PVR reduction was greater in the ISDN group, with a significant between‐group effect favouring ISDN (F(1,78) = 10.04, *p* = 0.002, partial *η* ^2^ = 0.114), whereas the time × group interaction was not significant, indicating parallel improvement trajectories (Table 2). The pre‐ and post‐treatment changes in PVR according to treatment group are illustrated in Figure 1. PSA levels remained stable over time in both groups, with no significant within‐ or between‐group differences (*p* > 0.75). ISDN was well tolerated, with only two patients reporting transient mild adverse effects and no serious complications (all categorized as Grade I according to the Clavien–Dindo classification^24^).

### 3.3 Predictors of successful TWOC

On univariate logistic regression analysis, treatment with ISDN plus standard therapy was significantly associated with successful TWOC (OR 3.44, 95% CI 1.31–9.06; *p* = 0.012). Age showed a non‐significant trend toward association, whereas disease duration, baseline PVR and baseline PSA were not predictive of outcome. In multivariable analysis, ISDN therapy remained an independent predictor of TWOC success (adjusted OR 3.27, 95% CI 1.23–8.70; *p* = 0.017; Table 3).

**TABLE 3:** Univariate logistic regression analysis of factors associated with successful trial without catheter (TWOC).

Variable	Univariate		Multivariate	
	OR (95% CI)	*p*‐value	OR (95% CI)	*p*‐value
Age (years)	1.05 (0.99–1.11)	0.129	1.04 (0.98–1.10)	0.202
Disease duration (days)	1.00 (1.00–1.01)	0.281	—	—
Baseline PVR (mL)	1.00 (1.00–1.00)	0.319	—	—
ISDN + standard therapy (vs. standard therapy)	3.44 (1.31–9.06)	**0.012**	3.27 (1.23–8.70)	**0.017**
Baseline PSA (ng/mL)	0.94 (0.58–1.51)	0.784	—	—

*Note:* Significant *p*‐values (<0.05) are visualized in bold.

Abbreviations: ISDN, isosorbide dinitrate; PSA, prostate‐specific antigen; PVR, post‐void residual.

## 4 DISCUSSION

AUR secondary to BPH remains a significant urological emergency, with substantial morbidity, a need for catheterization and potential progression to surgical intervention. The present study demonstrates that adjunctive sublingual ISDN enhances the efficacy of standard medical therapy—tamsulosin with or without finasteride—by improving both TWOC success rates and PVR volumes. Patients receiving ISDN exhibited a TWOC success rate of 50.0% compared with 22.5% in the control group, alongside a markedly reduced mean PVR (82.7 ± 9.8 mL vs. 171.6 ± 72.3 mL). These improvements were achieved without clinically significant adverse events, with only two patients reporting mild, transient effects (categorized as Grade I according to the Clavien–Dindo classification^24^).

These findings align with and extend prior evidence on NO donors in managing lower urinary tract symptoms (LUTS) and AUR. Consistent with Tadayyon et al.,^22^ who reported a 30% spontaneous voiding success with sublingual ISDN versus 3.3% in controls (*p* = 0.006), our results demonstrate enhanced TWOC outcomes with this approach. Similarly, Roshani et al.^25^ observed significant reductions in residual urine volume following single‐dose sublingual ISDN, supporting the hypothesis that NO‐mediated smooth muscle relaxation in the bladder neck and urethral sphincter facilitates voiding in obstructive uropathy. However, our findings contrast with Golikhatir et al.,^23^ who found a numerically higher but not statistically significant improvement in TWOC success rates when adding extended‐release ISDN to tamsulosin (81.6% vs. 67.5%, *p* = 0.155). These divergent results may reflect differences in drug formulation, dosing regimen or timing of administration. The current study employed sublingual ISDN at 10 mg daily for 21 days, whereas Golikhatir et al.^23^ utilized a higher oral dose (40 mg extended‐release) for a shorter duration (3 days). The route of administration may be particularly relevant, as sublingual delivery bypasses first‐pass metabolism and may achieve more rapid and reliable therapeutic concentrations.

Mechanistically, ISDN releases NO, activating sGC and increasing cGMP in smooth muscle cells, resulting in relaxation of urethral and bladder neck smooth muscle.^18^, ^19^, ^26^ This pharmacological effect complements α1‐adrenergic antagonists like tamsulosin, which primarily alleviate dynamic bladder outlet obstruction. The observed improvement in TWOC success and PVR reduction likely reflects this synergistic mechanism, though the specific contribution of ISDN versus tamsulosin alone cannot be definitively isolated given the study design.

The current study also extends the literature by providing comprehensive outcome data beyond acute voiding outcomes. Unlike several previous investigations that focused solely on acute outcomes (Tadayyon et al.^22^; Golikhatir et al.^23^), the present study assessed PVR volumes and PSA levels after a 21‐day treatment period. The significant reduction in PVR aligns with prior combination therapy studies (Singh et al.^27^), though those studies focused on tamsulosin plus tadalafil rather than ISDN, emphasizing the novelty of our findings.

The 21‐day daily sublingual ISDN regimen should be interpreted with caution, as prolonged nitrate exposure may induce tolerance, managed by nitrate‐free intervals,^26^ which were not applied in the present study. Importantly, the mechanisms underlying nitrate tolerance have not been established in bladder tissue, limiting direct extrapolation from cardiovascular research. In a related context, although catheterization for less than 3 days appears to be safe,^28^ recent meta‐analysis suggests that neither immediate nor delayed TWOC modifies the likelihood of successful spontaneous micturition.^29^

Regarding safety, the combination of ISDN with α1‐blockers warrants particular attention given the increased risk of hypotension. The present study demonstrates a favourable profile for sublingual ISDN, with only two patients experiencing mild headache and resolved promptly with oral acetaminophen, with no drug discontinuations. This contrasts with Tarhan et al.,^30^ who reported a higher incidence of mild adverse events including headache, dizziness and hypotension with isosorbide mononitrate. The difference in adverse event rates may relate to the lower dose (10 mg vs. 60 mg) and sublingual administration route used in the current study. Importantly, the safety profile of ISDN in this context appears comparable to that of PDE5 inhibitors, which commonly cause headache, flushing and hypotension (Singh et al.^30^; Bechara et al.^31^).

The findings of this study have to be seen in light of some limitations. The sample size, although adequately powered, remains relatively modest and may restrict the detection of less common adverse events or subgroup effects. The 21‐day follow‐up, although sufficient for acute outcomes, limits inference regarding long‐term efficacy, recurrence of AUR, or effects on prostate volume and bladder function. This study was limited by the absence of a placebo arm, which could potentially confound the outcomes. The lack of data on certain confounding variables (including bladder wall thickness, concomitant hydronephrosis and prostate volume) may affect the accuracy of the results and should be considered in future research. Additionally, the study did not incorporate quality‐of‐life measures, which would be valuable for assessing the patient‐centred impact of the intervention. Finally, the study was conducted at a single centre, limiting generalizability to other healthcare settings.

Future research should focus on longer‐term, multicentre studies to evaluate durability of response, recurrence rates and optimal dosing strategies. Comparative trials with other adjunctive therapies would clarify relative efficacy, whereas inclusion of patient‐reported outcomes would allow a holistic assessment of clinical benefit.

## 5 CONCLUSION

Adjunctive sublingual ISDN represents a viable therapeutic approach to improve spontaneous voiding, reduce residual urine and potentially decrease the need for prolonged catheterization or surgical intervention. From a health system perspective, this approach may reduce emergency department utilization, hospital admissions and associated expenses. The results further reinforce the NO–cGMP pathway as a mechanistic target in LUTS management, supporting multi‐targeted approaches that address both dynamic and static components of bladder outlet obstruction.

## AUTHOR CONTRIBUTIONS

**Salman Soltani:** Conceptualization; methodology; investigation; resources; project administration; supervision; writing—review and editing. **Hamid Reza Ghorbani:** Conceptualization; methodology; investigation; resources; project administration; supervision; writing—review and editing. **Mahmoud Tavakkoli:** Investigation; resources; writing—review and editing. **Fatemeh Sadabadi:** Writing—original draft; writing—review and editing. **Seyyed Parham Ahmadi:** Formal analysis; visualization; writing—original draft; writing—review and editing. **Farid Zeinali:** Investigation; resources; writing—review and editing. All authors read and approved the final version of the manuscript.

## CONFLICT OF INTEREST STATEMENT

The authors declare no conflicts of interest.

## Supporting information


**Figure S1.** CONSORT flow diagram: Flow of participants through each stage of the study progress.

## Data Availability

The data that support the findings of this study are available from the corresponding author upon reasonable request.
