# Supplementary figures and images for: Isosorbide dinitrate as an effective adjunct therapy for acute urinary retention: A randomized controlled trial
Source: BJUI Compass. 2026 Jul 27;7(7):e70252. doi: 10.1002/bco2.70252 (PMC13407321; doi:10.1002/bco2.70252)

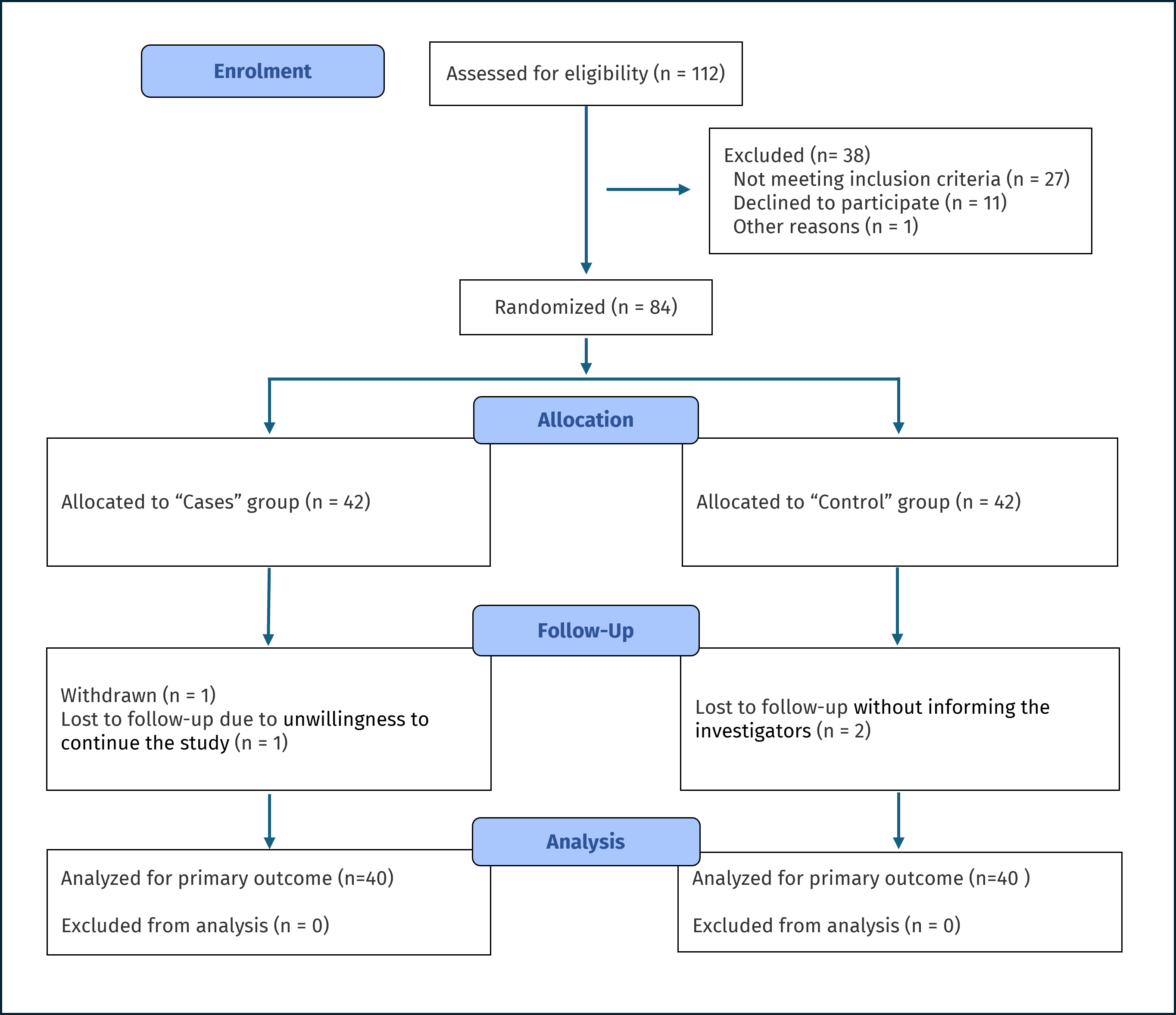

Supplement: Supplementary file 1 — Figure S1. CONSORT flow diagram: Flow of participants through each stage of the study progress. [file BCO2-7-e70252-s001.png]
